# Multi-Scale Modeling of HIV Infection *in vitro* and APOBEC3G-Based Anti-Retroviral Therapy

**DOI:** 10.1371/journal.pcbi.1002371

**Published:** 2012-02-09

**Authors:** Iraj Hosseini, Feilim Mac Gabhann

**Affiliations:** Institute for Computational Medicine and Department of Biomedical Engineering, Johns Hopkins University, Baltimore, Maryland, United States of America; Eötvös Loránd University, Hungary

## Abstract

The human APOBEC3G is an innate restriction factor that, in the absence of Vif, restricts HIV-1 replication by inducing excessive deamination of cytidine residues in nascent reverse transcripts and inhibiting reverse transcription and integration. To shed light on impact of A3G-Vif interactions on HIV replication, we developed a multi-scale computational system consisting of intracellular (single-cell), cellular and extracellular (multicellular) events by using ordinary differential equations. The single-cell model describes molecular-level events within individual cells (such as production and degradation of host and viral proteins, and assembly and release of new virions), whereas the multicellular model describes the viral dynamics and multiple cycles of infection within a population of cells. We estimated the model parameters either directly from previously published experimental data or by running simulations to find the optimum values. We validated our integrated model by reproducing the results of *in vitro* T cell culture experiments. Crucially, *both* downstream effects of A3G (hypermutation and reduction of viral burst size) were necessary to replicate the experimental results *in silico*. We also used the model to study anti-HIV capability of several possible therapeutic strategies including: an antibody to Vif; upregulation of A3G; and mutated forms of A3G. According to our simulations, A3G with a mutated Vif binding site is predicted to be significantly more effective than other molecules at the same dose. Ultimately, we performed sensitivity analysis to identify important model parameters. The results showed that the timing of particle formation and virus release had the highest impacts on HIV replication. The model also predicted that the degradation of A3G by Vif is not a crucial step in HIV pathogenesis.

## Author Summary

According to UNAIDS (The Joint UN Programme on HIV/AIDS) and WHO, HIV/AIDS has killed more than 25 million people worldwide since its recognition in 1981. Recently, APOBEC3G, a member of the APOBEC family, has been shown to be a potent inhibitor of HIV infection. In contrast, a viral protein, called Vif, is known to protect the virus by binding to APOBEC3G and causing the degradation of this enzyme. We have developed a computational model to simulate *in vitro* experiments that include A3G-Vif interactions at the intracellular level and T cell-HIV dynamics at the multicellular level. Experimental data were used to establish system parameters and also to validate predictions of our models. We studied various drugs targeting APOBEC3G and Vif pathways to find the optimum therapeutic approach against HIV replication. Our model predicted that a mutated form of APOBEC3G that does not bind to Vif performs significantly better at suppressing HIV replication compared to other drugs. We also found that the drug should be administered shortly after infection and it must be available to all cells in order to be effective.

## Introduction

Over the past decade, some human innate restriction factors have been found to attenuate viral replication. These restriction factors, including human APOBEC3G (apolipoprotein B mRNA editing enzyme, catalytic polypeptide-like 3G, or A3G), a potent inhibitor of human immunodeficiency virus type 1 (HIV-1) infection, have been extensively reviewed in [1]–[6] among others. A3G, a member of the APOBEC family, counteracts retroviral infection primarily by hypermutating retroviral cDNA and by inhibition of viral reverse transcription and integration. In a HIV-infected cell, A3G produced by the cell is encapsulated in progeny HIV-1 particles by binding to the viral RNA genome. When these viruses are released and infect another cell, A3G causes excessive C-to-U deamination of the minus strand DNA during reverse transcription [7]–[11]. This results in G-to-A hypermutations in the plus strand cDNA [7]–[9], [12] with a mutational frequency of over 10% [2], [13]. It has also been proposed that uracil-DNA glycosylases, such as UNG2 or SMUG1 may trigger degradation of uracilated minus strand DNA [14], [15]. But, some reports showed that uracil DNA glycosylases do not contribute to antiviral activity of A3G 16–18.

It has been suggested that hypermutation may not be the only A3G activity that restricts HIV replication [19], [20]. Deaminase-independent activities of A3G include, but are not limited to, inhibiting synthesis of viral cDNA by blocking translocation of reverse transcriptase along the template RNA [21]–[23], reduction in the ability of tRNA^Lys3^ primers to initiate reverse transcription [24], [25], blocking integration of the double-stranded viral DNA by causing defects in cleavage of tRNA^Lys3^ primer [18], or inhibiting nuclear import of pre-integration complex [26]. Although there is mounting evidence for deaminase-independent activities of A3G, several reports have suggested that these activities are the results of over-expression of A3G in cells [27]–[29]. As mentioned, A3G normally mediates antiviral activities in the target cells after being packaged in the newly budded viruses from the virus-producing cells. Evidence supporting this observation came from studies performed almost 10 years before discovery of A3G [30]–[32]. Recently, Chiu *et al.* have shown that A3G may also restrict HIV-1 infection in resting CD4+ T cells [33]; however, other groups could not verify this phenomenon [34], [35]. Further investigation is required to illuminate the new potential mechanisms of A3G against HIV-1.

**Figure 1:**
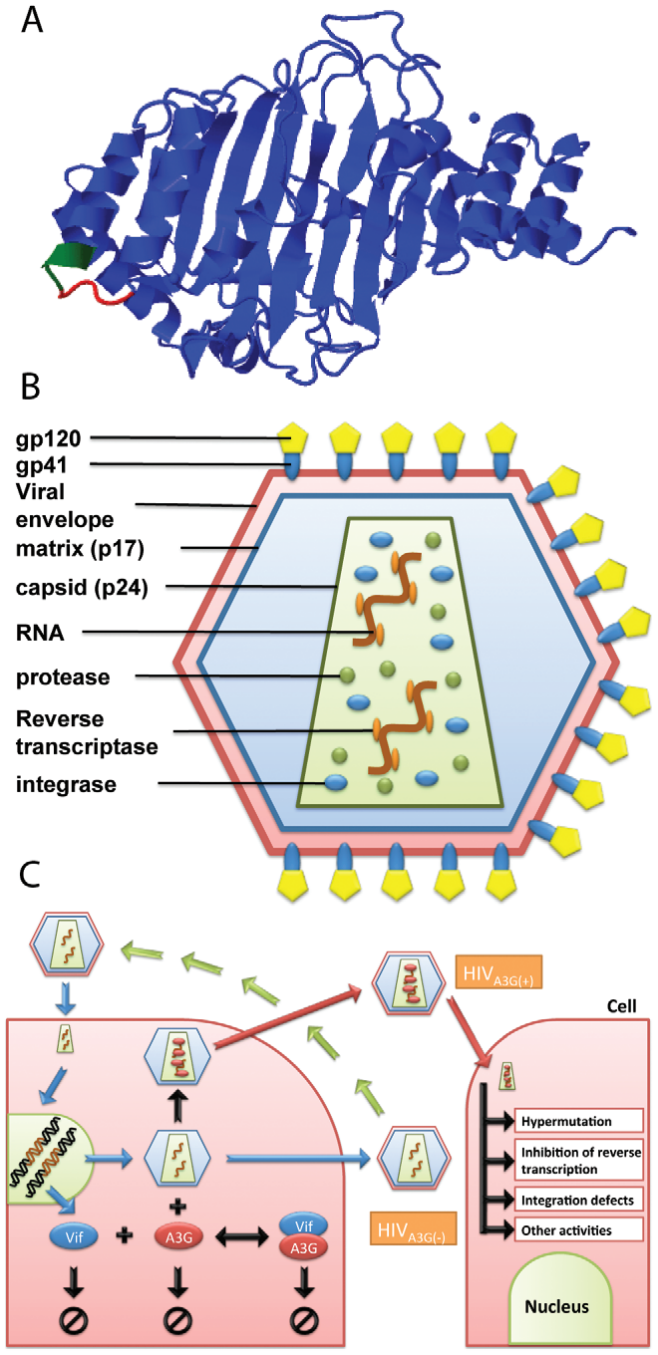
APOBEC3G 3D model structure, HIV virion and its life cycle. (A) 3D model structure of A3G proposed by Zhang *et al.* [47]. The 124–127 motif (red) is located beside the 128–130 Vif-binding region (green). (B) HIV particles are surrounded by fatty materials known as the viral envelope. The matrix formed from p17 protein is another layer underneath the viral envelope. The particles also contain two exact copies of RNA strands as well as three essential enzymes required for replication: reverse transcriptase, integrase and protease. (C) Mechanism of HIV infection including viral entry, genome integration, production and release of new viral particles, and encapsulation of A3G into virions is schematically shown. If the released viruses carry A3G, they are denoted A3G(+) viruses, otherwise they are denoted A3G(−). When A3G(+) viruses infect the next cell, the packaged A3G has several activities such as hypermutating the minus strand of viral DNA, and inhibiting various steps of reverse transcription and integration. “Null” symbols inside the cell represent degradation of Vif, A3G, and A3G-Vif complex.

HIV-1 is a retrovirus, more specifically a lentivirus. It encodes nine genes, of which *gag*, *pol* and *env* are common to all retroviruses. The remaining 6 genes (*tat*, *rev*, *vif*, *vpr*, *vpu* and *nef*) encode proteins with accessory and/or regulatory roles crucial to HIV pathogenesis [36], [37]. Viral infectivity factor (Vif), a 23-kDa protein, has an important function in HIV infectivity by inhibiting A3G. It is thought that Vif's primary mechanism of action is to deplete the intracellular pool of A3G by inducing polyubiquitylation and eventual degradation of A3G through the proteasomal pathway [11], [38]–[41]. Recently, it has been shown that A3G N terminal is a target of Vif-induced polyubiquitylation [42]. However, some evidence suggests that Vif more directly impedes A3G incorporation into HIV-1 virions [12], [43]. Sequence analysis studies have shown that an Asp-Pro-Asp motif at positions 128–130 in A3G is crucially important for binding of Vif to A3G. The D128K mutation in A3G protects the protein from Vif-induced degradation [12], [44], [45]. Mutations at Tyr-124 or Trp-127 make the protein unable to bind viral RNA and therefore get packaged into viruses [46]–[48]. The 124–127 motif is located beside the 128–130 Vif-binding region in the 3D model structure of A3G shown in Fig. 1A [47]. This suggests that Vif binding and RNA binding may be in competition.

Mathematical models have proven valuable in understanding the dynamics of HIV-1 infection *in vivo* [49]. In most existing HIV infection models, subcellular events such as viral genome replication and integration, production of viral proteins, and release of new virions are often not explicitly reflected [50]–[56]. Instead, these extracellular models consider several cycles of infection where a population of cells can be infected by viruses and the effects of drug therapies on the number of viruses are studied. By contrast, intracellular models assuming only a single cycle of infection have been limited to the study of the kinetics of virus and host proteins and their interactions to understand the dynamics of viral replication inside the cells [57], [58]. Both types of modeling give insights into how HIV disease progresses in the body, however, combining intracellular and extracellular models would greatly enhance our understanding in this area [59].

In our previous work [60], we used a model of a single cell that could undergo multiple re-infection as a surrogate for multicellular infection, to capture both intracellular and extracellular properties of HIV infection. In the present study, we have developed a multi-scale system integrating intracellular, cellular and extracellular processes. This integrated model explicitly includes concepts such as burst size (the number of viruses released by a cell), proliferation rate of cells, cell life cycle, virus clearance, and intracellular delays in viral formation and release from cells, which were not explicitly described in the previous model. The integrated model is used to simulate *in vitro* T cell HIV infection experiments.

The intracellular (single-cell) model includes interactions between Vif, virus RNA and human A3G. Experimental data are used to establish system parameters such as degradation rate constants of proteins, life-span of infected cells, and viral generation time. The intracellular model sheds light on how changes in the intracellular parameters affect the production and release of new HIV viruses. The single-cell model results are integrated into a multicellular model to simulate T cell culture experiments. We estimate certain parameters such as viral burst size, HIV infectivity rate, and virus clearance rate using experimental data, and model predictions are verified using previously published experimental results. Biologically relevant levels of host and virus proteins in experiments are estimated using our multi-scale system. We monitor how the population of cells acts in response to virus infection. Several drugs targeting A3G and Vif pathways are studied to compare their efficacy at different doses. We also estimate drug efficacy under non-ideal conditions, such as when it is available to only a specific fraction of cells in the whole population, or delivered at later times following infection.

## Methods

### HIV Biology and System Model

HIV particles are surrounded by a fatty membrane known as the viral envelope. There is another layer underneath the viral envelope called matrix which is formed from p17 protein. HIV has three essential enzymes required for replication: reverse transcriptase, integrase and protease. These enzymes along with two exact copies of RNA strands are packaged in the viral core or capsid. This is also where the A3G is encapsulated into the virus. The viral core is made from the protein p24. A generic structure of HIV virus is shown in Fig. 1B.

Fig. 1C shows the schematic model of HIV infection that is used to develop the computational model, capturing both intracellular and extracellular information. Each cycle of infection begins with a HIV virus attacking a healthy normal cell. After the virus entry into cells, the HIV genome is reverse transcribed into cDNA [61]. Some evidence suggests that HIV capsid remains intact during reverse transcription and that uncoating occurs at the nuclear pore upon completion of reverse transcription, reviewed in [62]. The resulting double-stranded DNA enters the nucleus along with the viral integrase, which splices the HIV DNA into the human genome. The integrated viral DNA, called provirus, is then transcribed into messenger RNA used as a blueprint for making new HIV proteins and enzymes. Some of the viral RNA remains as full-length RNA copies, to be incorporated as viral genetic material for new virions. We model the mechanisms from virus entry to viral protein production using relevant kinetics and intracellular delay parameters. The focus of this study is the interaction between Vif and A3G, and their productions are explicitly included in our model.

HIV enzymes, structural proteins, and full-length RNA molecules are assembled into virions at the cell membrane. Human T cells can produce A3G as an intrinsic defense mechanism. This protein binds the viral RNA and gets encapsidated into the viral capsids while they are still inside the cell. Shortly after viral assembly, the viruses get released from the cells and they are ready to infect new cells. In our model, if the released viruses carry A3G, they are denoted A3G(+) viruses, otherwise they are denoted A3G(−). The encapsulated A3G is assumed to not have effects on viral entry. This is simply because the entry process involves the binding of CD4 and chemokine coreceptors on the T cell surface to gp41 and gp120 on the viral envelope whereas A3G is encapsulated inside the capsid and doesn't interact with the proteins on the viral envelope. When A3G(+) viruses infect the next cell, the packaged A3G can have various anti-retroviral activities. In this paper, we focus on two downstream effects of A3G; 1) hypermutation in the minus strand of viral DNA; and 2) inhibition of viral cDNA production. This means that even though A3G(+) viruses can infect cells with similar rate of infectivity to A3G(−) viruses, infected cells produce fewer virions. HIV has evolved to combat A3G with Vif. The Vif protein binds A3G and facilitates its polyubiquitylation, and therefore increases its degradation rate. This Vif-induced degradation, and basal rates of degradation of both Vif and A3G, are included in the model. Note that we assume A3G doesn't affect replication of viruses in the producer cell. This has been observed in [30]–[32] and can be explained assuming that reverse transcription occurs inside the capsid and A3G doesn't have access to transcripts. The single-cell and multicellular models of HIV infection are described using differential equations.

### Mathematical Model Development for Intracellular Interactions (“Single-Cell Model”)

Our model includes both proteins and virions in a generic human T cell. There is a differential equation for each entity, which describes its production, degradation, and interactions with other entities.

**Figure 2:**
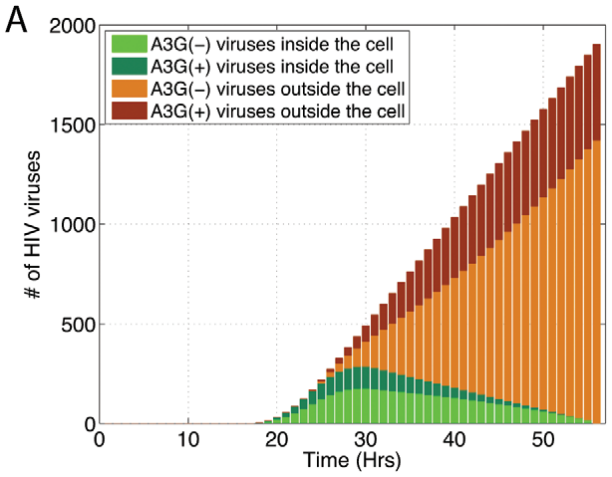
Time-dependent profile of virus release from a single cell. The time evolution of total number of A3G(−) and A3G(+) viruses produced inside a single cell and released from it is shown. Infection occurs at *t* = 0. Production of new viruses begins approximately 16 hours after infection. Light and dark green colors represent A3G(−) and A3G(+) viruses inside the cell. Newly produced viruses get released to the extracellular environment at 22 hours post infection and the virus release continues until the cell dies approximately 55 hours after infection. A3G(−) and A3G(+) viruses outside the cell are represented by light and dark brown colors.

The A3G protein can be produced, degraded, and incorporated into progeny viruses. It also binds to and dissociates from Vif.
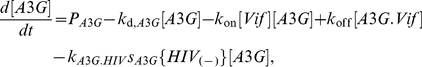
(1)where *P_A3G_* is the production rate of A3G, *k*_d,*A3G*_ is the degradation coefficient of A3G, *k*_on_ and *k*_off_ are the binding and dissociation constants of the A3G-Vif complex, and *k_A3G.HIV_* is the rate constant for A3G incorporation into A3G(−) HIV viruses, denoted by *HIV*_(−)_. The stoichiometry of A3G proteins incorporated into virions is *s_A3G_*. Similarly, Vif concentration is governed by
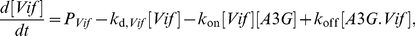
(2)where *P_Vif_* and *k*_d,*Vif*_ are the production rate and the degradation coefficient of Vif, respectively. The A3G-Vif complex can be formed from Vif binding to A3G or it can degrade.
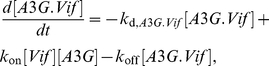
(3)In (3), the degradation coefficient of A3G-Vif complex is shown by *k*_d,*A3G.Vif*_. The number of HIV virions inside the cell is also modeled by
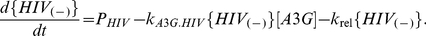
(4)Viruses are produced at a rate of *P_HIV_* and budded off from the cell by a rate of *k*_rel_. The A3G protein can get encapsulated into A3G(−) viruses and convert them to A3G(+) viruses. The number of intracellular A3G(+) viruses is governed by
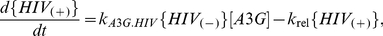
(5)where *HIV*_(+)_ refers to A3G(+) viruses. Finally, the release of newly-made HIV viruses is described by the following equations.
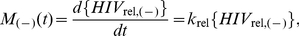
(6a)

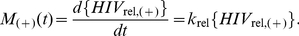
(6b)In equation (6), *HIV*_rel,(−)_ and *HIV*_rel,(+)_ represent released A3G(−) and A3G(+) viruses, respectively. *M*_(−)_(*t*) and *M*_(+)_(*t*), the number of released A3G(−) and A3G(+) viruses at time *t*, will be used later in the extracellular model. In the model, proteins are quantified in units of molar concentration, whereas viruses are quantified as discrete numbers of viral particles. Fig. 2 shows the time evolution of total number of A3G(−) and A3G(+) viruses produced in a single cell after infection and released from it to the extracellular environment.

### Mathematical Model Development for Extracellular Events (“Multicellular Model”)

**Figure 3:**
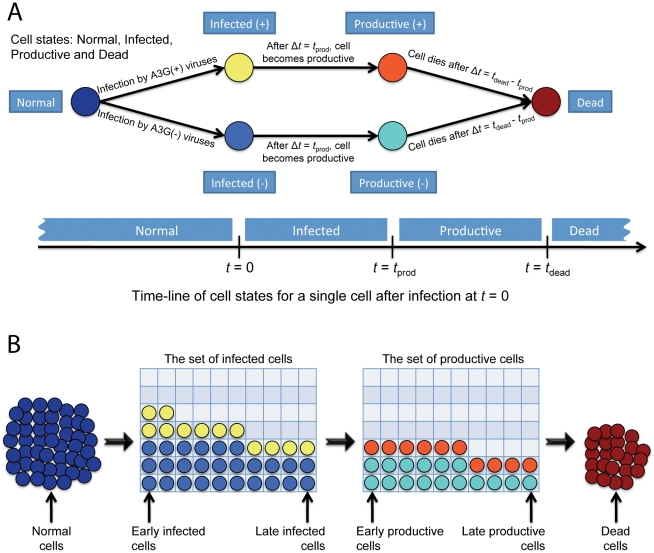
A schematic diagram of cell states and a snapshot of the multicellular model. (A) each cell lives in the “Normal” state until a HIV virus infects it. An “Infected” cell doesn't release new virions until a certain time point post infection, denoted *t*_prod_. At this time point, the cell becomes “Productive” and begins releasing viruses into the extracellular environment until it dies at *t*_dead_, when it is marked as “Dead”. The “Infected(+)” and “Infected(−)” states correspond to cells that have been infected by A3G(+) and A3G(−) viruses, respectively. The same concept applies to “Productive(+)” and “Productive(−)” cells. (B) The time of infection is known for each cell in our multicellular model. A snapshot of the multicellular model shows cells with different post-infection ages in the sets of infected and productive cells. Normal cells become infected and enter the set of infected cells as early-infected cells. The late-infected cells become productive and leave the set of infected cells to join the set of productive cells where they are shown as early-productive cells. Finally, late-productive cells die, exit the set of productive cells, and get marked as dead.

Our multicellular model describes an extracellular pool of HIV viruses infecting a population of T cells, specifically, in cell culture. This model includes cellular and extracellular properties including the production rate of T cells, rate of infection by HIV viruses, variations in levels of A3G(−) and A3G(+) viruses, and burst size (which is defined as the average number of HIV viruses made by an infected cell). There is a strong link between the intracellular and multicellular models through the burst size and the release distribution of A3G(−) and A3G(+) viruses over time. The multicellular model can be described by a set of equations and constraints. In our model, we define *T*_0_ as the initial number of “Normal” T cells. Each cell lives in the normal state until a HIV virus infects it. “Infected(+)” and “Infected(−)” states correspond to cells that have been infected by A3G(+) and A3G(−) viruses, respectively; however, infected(+) cells produce fewer viruses than infected(−) cells. It is assumed that there is no hyper-infection, that is, after a virus attacks and enters a healthy cell, the cell becomes infected, CD4 is down-regulated [63], and no more viruses attack it. Cells in infected(+) and infected(−) states become “Productive(+)” and “Productive(−)” after Δ*t* = *t*_prod_ post infection, respectively and begin releasing viruses into the extracellular environment. The release continues until Δ*t = t*_dead_ after infection, when the cell dies and it is marked “Dead”. A schematic diagram of cell states is shown in Fig. 3A.

Fig. 3B is a snapshot of the multicellular model at a specific time, showing cells of different post-infection ages at different states. In our simulations, we keep time of infection for each cell in the multicellular model. This is represented by early and late infected cells in the set of infected cells and by early and late productive cells in the set of productive cells.

The number of healthy and infected cells in our model is governed by the following equations.
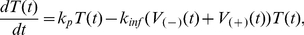
(7)and
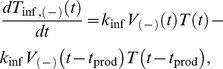
(8a)

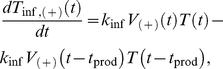
(8b)where *T* is the number of healthy cells and the rate of infection is defined by *k*_inf_. The proliferation rate of healthy cells is represented by *k_p_* = ln(2)/*t_T_*_,2_ where *t_T_*_,2_ is the cell doubling time. A3G(−) and A3G(+) viruses are denoted by *V*_(−)_ and *V*_(+)_, respectively, and *t* represents time post inoculation of the T cell culture. Note that *V*_(−)_(*t*), *V*_(+)_(*t*), and *T*(*t*) are all zero for t<0. In (7), the number of normal T cells increases by cell proliferation and decreases as cells get infected.

In (8a) and (8b), *T*_inf,(−)_ and *T*_inf,(+)_ represent the number of cells infected by A3G(−) and A3G(+) viruses, respectively. As mentioned earlier, each infected cell begins releasing new viruses after a time *t*_prod_ post infection. At this point, the infected cells become actively productive, represented by *T*_prod,(−)_ and *T*_prod,(+)_. Note that in (8a), there are two mathematical terms determining the rate of change for *T*_inf,(−)_. The first term, *K*_inf_ *V*_(−)_(*t*)*T*(*t*), represents the number of cells that become infected by A3G(−) viruses at time *t* and enter the set of infected cells, whereas the second term, *K*_inf_ *V*_(−)_(*t*−*t*_prod_)*T*(*t*−*t*_prod_), represents the number of cells that were infected at time *t*−*t*_prod_, i.e., they are productive at time *t* and leave the set of infected cells to join the set of productive cells (Fig. 3B). The mathematical terms in (8b) are the same as those in (8a), except that they deal with infected(+) cells. The number of productive cells is described by
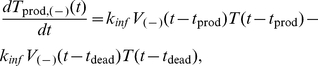
(9a)

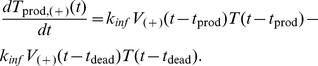
(9b)In (9a), the first term, *K*_inf_ *V*_(−)_(*t*−*t*_prod_)*T*(*t*−*t*_prod_), represents the number of cells that become productive at time *t* and enter the set of productive cells. Once the cells are infected with HIV, they have an average life span of *t*_dead_. This means that productive cells release HIV viruses from *t*_prod_ until their death at *t*_dead_. The second term in (9a), *K*_inf_ *V*_(−)_(*t*−*t*_dead_)*T*(*t*−*t*_dead_), represents the number of cells that were infected at time *t*−*t*_dead_, i.e., they are dead at time *t* and leave the set of productive cells (Fig. 3B). The mathematical terms in (9b) are the same as those in (9a), except that they describe productive(+) cells. The number of dead cells is represented by *T*_dead_, governed by
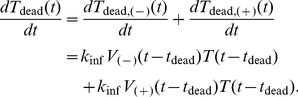
(10)Along with the equations for different cell states, the model tracks extracellular viruses.
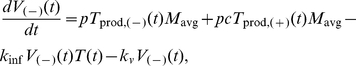
(11a)

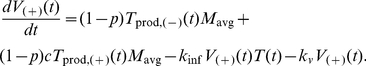
(11b)The average number of viruses released from a productive(−) cell is *M*_avg_ = *B*/(*t*_dead_−*t*_prod_) where *B* is the viral burst size. The percentage of released viruses that do not contain A3G is denoted by *p*. The encapsulated A3G in the HIV virus has anti-viral activities in the target cell. This results in that a productive(+) cell produces fewer viruses than a productive(−) cell. The reduction in burst size of productive(+) cells is denoted by *c*. Both *p* and *c* take values between 0 and 1. In (11a), the first and second terms refer to the number of A3G(−) viruses being produced from productive(−) and productive(+) cells, whereas the third term represents the number of viruses that are infecting cells. The last term shows the number of viruses that are being cleared from the culture. The clearance rate is represented by *k_v_* = ln(2)/*t_v_*_,1/2_ where *t_v_*_,1/2_ is HIV half-life *in vitro*. Mathematically similar terms describe the number of A3G(+) viruses in (11b).

Parameters *p* and *c* play important roles in our simulation. Since *p* has a direct effect on the shape of HIV replication curves (described later), we call this parameter HIV replicative potential. The value of *p* inversely correlate with intracellular A3G getting encapsulated in newly made viral particles. It is desirable for both *p* and *c* to have values as close as possible to zero to efficiently stop HIV replication. Nominal values of *p* and *c* are shown in Table 1 for different types of viruses and cells. Note that *p* is a property of cells whereas *c* is a property of viruses. To compute the number of A3G(−) and A3G(+) viruses in (11a) and (11b), we assumed that the release rate of viruses from a productive cell over period of [*t*_prod_, *t*_dead_] is constant and also *p*, the HIV replicative potential remains constant during this period.

**Table 1:** Values of *p* and *c* for different cases of viruses and cells.

	A3G(−) viruses	A3G(+) viruses
**Target cells express A3G**	p = Low, c≈1	p = Low, c≈Low
	Output: A3G(−) and A3G(+) viruses	Output: A3G(−) and A3G(+) viruses
**Target cells do not express A3G**	p≈1, c≈1	p≈1, c≈Low
	Output: only A3G(−) viruses	Output: only A3G(−) viruses

As we will see in the results section, the release rate of viruses from a productive cell is not constant at all times. In fact, virus release begins at *t*_prod_ post infection, increases for 8 hours and remains constant until the cell dies. Also the ratio of released A3G(−) viruses to total released viruses is not constant during the virus release period and changes over time as it can be seen in Fig. 2. Therefore we use the following equations instead of (11a) and (11b) to accurately compute the number of viruses without having any assumptions on the release of viruses.
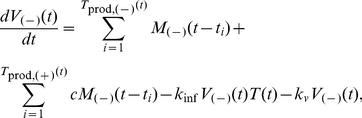
(12a)

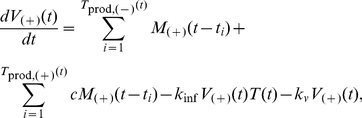
(12b)
*M*_(−)_(*t*) and *M*_(+)_(*t*) are the number of released A3G(−) and A3G(+) viruses from a single cell at time *t* after infection, defined in (6a) and (6b). The first term in (12a) is a summation over all the A3G(−) viruses that are being released at time *t* from all the cells in the set of productive(−) cells. The time of infection for the *i*th cell in the set is denoted by *t_i_*. The second term in (12a) is similar to the first term, but deals with the A3G(−) viruses released from the productive(+) cells. Similar mathematical expressions are used for computing the number of A3G(+) viruses in (12b).

Viruses in cell culture can become non-infectious or dead after some time. The number of cleared viruses from the culture is given by
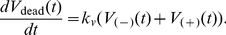
(13)


### Integration of Single-Cell Model Results into the Multicellular Model

As noted above, the single cell and multicellular models are linked through the burst size and the release distribution of A3G(−) and A3G(+) viruses over time. In this paper, we use the following two methods to establish this link.

In the first method, the multicellular model assumes that release rate of viruses from a productive cell over period of [*t*_prod_, *t*_dead_] is constant and also that the ratio of released A3G(−) viruses to total released viruses at each time point during virus release is constant and equal to *p*. With these assumptions, *V*_(−)_(*t*) and *V*_(+)_(*t*) can be easily computed using (11a) and (11b). In the second method, the multicellular model makes no assumption on the rate of virus release and uses the actual time-dependent profile of virus release from a single cell to compute the total number of A3G(−) and A3G(+) viruses in culture supernatant by using (12a) and (12b). Although this method provides a comprehensive link between the two models, it would be difficult to optimize the system parameters. Therefore, we use the first method for optimizing the single-cell and multicellular model parameters. Having done that, we use the second method for our simulations regarding effects of A3G-based therapeutic strategies, drug penetrance and administration time on HIV replication.

## Results

### Single-Cell Model of APOBEC3G-Vif Interactions

**Figure 4:**
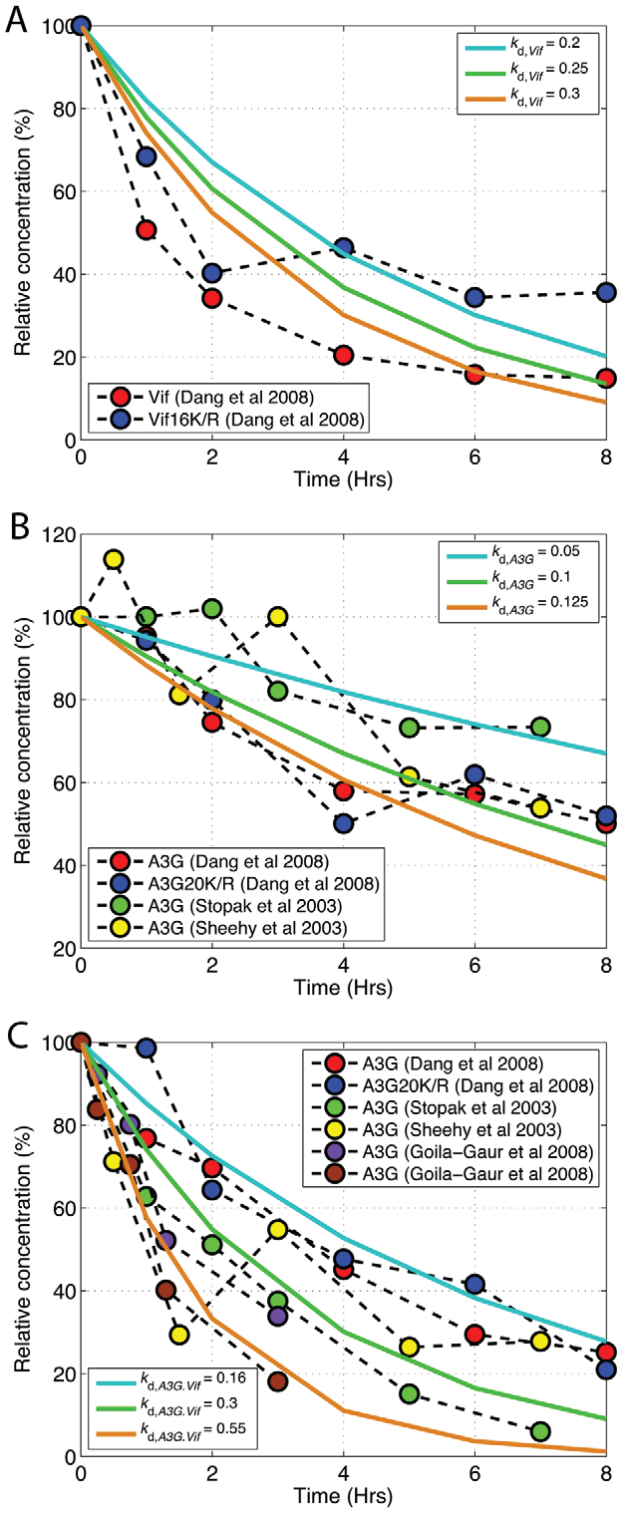
Degradation profile of protein entities in the model. Using experimental data and first-order decay curves, degradation coefficients of (A) Vif, (B) A3G, and (C) A3G-Vif complex were estimated to be 0.25 hr^−1^, 0.1 hr^−1^, and 0.3 hr^−1^, respectively (re-plotted from Benedict *et al.* [60]).

For our simulations, we obtained parameters from published biological experiments. Several groups have measured the degradation profiles of Vif and of A3G in the presence and absence of Vif [38], [41], [64], [65]. First-order kinetic decay curves were used to approximate the degradation rate of Vif, A3G, and A3G-Vif complex from this experimental data as 0.25 hr^−1^, 0.1 hr^−1^, and 0.3 hr^−1^, respectively (Figs. 4A–C). The binding affinity of A3G to Vif has been estimated to be in the low micromolar range by surface plasmon resonance [66]. We assume a value of 1 µM for the binding affinity, and calculate *k*_on_ by assuming *k*_off_ = 3600 hr^−1^. The stoichiometry of A3G and HIV, i.e., the number of A3G proteins incorporated into a virion, *s_A3G_*, has been estimated at seven molecules [67]. In an infected cell, viral protein production does not start as soon as HIV enters in the cell. Based on published experimental studies, Vif production begins approximately 12 hours after infection (*t*_prod,*Vif*_ = 12 hr), increases through 24 hours (Δ*t*_rise,*Vif*_ = 12 hr) and remains roughly constant after that. This was experimentally observed by measuring expression of HIV-1 RNA transcripts during HIV infection in [68], [69] and computationally verified in [57]. HIV particle formation is assumed to begin 16 hours post infection (*t*_form,HIV_ = 16 hr), continue to increase for 8 hours (Δ*t*_rise,HIV form_ = 8 hr) and plateau after that. In addition, a 6-hour delay is assumed for the budding process, meaning that virus release begins at 22 (*t*_rel,HIV_ = 22 hr) and increases through 30 hours post infection (Δ*t*_rise,HIV rel_ = 8 hr). These assumptions are consistent with measurements of reverse transcriptase activity in cell culture supernatants indicating active release of viruses from cells [69] and with the predictions of theoretical modeling [57].

Using the above parameters as a basis, we estimated the production rates of A3G and Vif. We have not found experimental data quantifying these production rates, therefore, we estimate these parameters using data from [11]. In those experiments, 293T cells were used as a ‘permissive’ cell line, meaning that they did not express A3G intrinsically. These cells were co-transfected with two vectors: one either a wild-type (WT) or a Vif-deficient (ΔVif) X4 provirus; the other (at varying doses) encoding A3G. After a day, the levels of supernatant p24 protein in the culture were monitored. The number of viruses can be calculated from this, as the 24-kDa p24 protein is estimated to be present at 2000–4000 molecule per virion [70], therefore 1 pg p24≈12,500 HIV particles. The supernatant including viruses from the infected 293T cells was extracted, normalized by p24 content, and used to challenge indicator cell lines in a single-cycle infection assay in which expression of chloramphenicol acetyltransferase (CAT) indicated HIV infection. After 28 hours, the number of infected cells were measured and normalized [11]. We use the single-cell model to find the percentage of released A3G(−) viruses in culture in the transfection part of this experiment and employ the multicellular model with equations (11a) and (11b) for the inoculation of CAT-indicator cells. Although the first part of this algorithm is a transfection of a T cell culture, information on the number of cells and transfection efficiency was not available. Moreover, we know that cells begin releasing viruses 22 hours after infection and in this experiment the culture supernatant was extracted 24 hours after transfection. This means that viruses in the supernatant were released from cells that were infected in the first two hours after transfection. Therefore, asynchronous infection would not be an issue in this case. This justifies using the single-cell model for the transfection part of this experiment.

**Figure 5:**
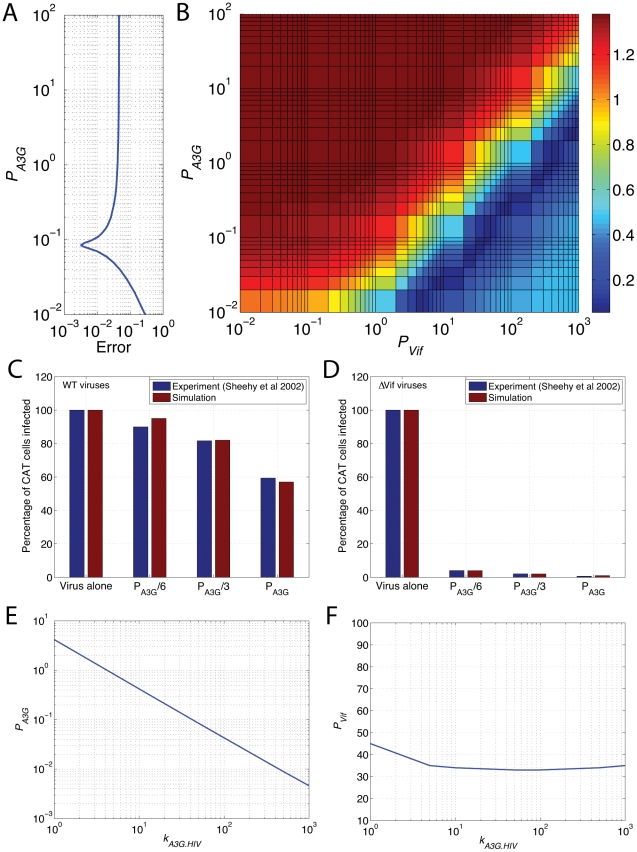
Estimation of A3G and Vif production rates. (A) shows the optimum *P_A3G_* for the ΔVif case (*P_Vif_* = 0), whereas (B) shows the fitness error heat-map for a wide range of values of (*P_Vif_*, *P_A3G_*) for the WT case. The error decreases as color changes from dark red to dark blue. The optimum *P_A3G_* can be read from (A) and projected to the dark blue region of (B) to find the optimum *P_Vif_*. The experimental data from Sheehy *et al.* [11] were used for estimating *P_Vif_* and *P_A3G_* is re-plotted as blue bars for (C) WT and (D) ΔVif viruses. The red bars show our predictions of percentage of CAT cells infected by using estimated *P_A3G_* and *P_Vif_* in our simulations. All the results in (A) and (B) are obtained for *k_A3G.HIV_* = 50 µM^−1^/hr. The optimum values of (E) *P_Vif_* and (F) *P_A3G_* versus *k_A3G.HIV_* are also shown.

Estimation of Vif and A3G production rates required an exhaustive search in *P_Vif_*-*P_A3G_* domain. The procedure is as follows. For a given pair of (*P_Vif_*, *P_A3G_*), the single-cell model computes the HIV replicative potential, the percentage of released viruses that are A3G(−). Next, in the multicellular model, CAT-indicator cells (*T*_0_ = 500,000 cells) are inoculated by viruses corresponding to 5 ng p24 with the ratio of A3G(−) to total viruses equal to *p*. The simulations are run for three different doses of A3G as in the experiment: (*P_Vif_*, *P_A3G_*), (*P_Vif_*, *P_A3G_*/3), and (*P_Vif_*, *P_A3G_*/6), and the number of infected cells at 28 hours is computed, normalized and compared to the experimental results to calculate the fitness error which is defined as the square root of sum of squares of differences between experimental data and computed results. For the HIV-ΔVif case (*P_Vif_* = 0 µM/hr), the error for a range of *P_A3G_* is depicted in Fig. 5A and the minimum error is achieved at *P_A3G_* = 0.085 µM/hr.

In contrast, Fig. 5B shows the error for the HIV-WT case for a wide range of values of *P_Vif_* and *P_A3G_*. The fitness increases from low to high as the color changes from red to dark blue. This figure gives us possible pairs of (*P_Vif_*, *P_A3G_*) producing best fits to the experimental data. Based on the value of *P_A3G_* obtained from Fig. 5A, the best fit is achieved at *P_Vif_* = 33 µM/hr. The experimental data from Sheehy *et al.* [11] used for calculating *P_Vif_* and *P_A3G_* is re-plotted as blue bars in Figs. 5C and 5D for WT and ΔVif viruses, respectively. The red bars show model predictions of percentage of CAT cells infected by using optimized *P_A3G_* and *P_Vif_* in our simulations.

The estimates of *P_A3G_* and *P_Vif_* were computed for an assumed value of *k_A3G.HIV_* = 50 µM^−1^/hr, because A3G incorporation rate is not known. We repeated the simulations for a range of *k_A3G.HIV_* values and estimated values of *P_A3G_* and *P_Vif_* with the minimum error are shown in Fig. 5E and Fig. 5F. We found that *P_A3G_* is inversely proportional with *k_A3G.HIV_*, whereas *P_Vif_* remains approximately constant. For the rest of this paper, we use the average value of *P_Vif_*, which is equal to 35.6 µM/hr. For the estimation of *P_Vif_* and *P_A3G_*, optimal values for burst size, infectivity rate and HIV half-life *in vitro* were used in the multicellular model. These parameters will be discussed in the next section.

### Multicellular Model of HIV Propagation in Culture

For the HIV replication experiments that we are simulating in this section, unlike the single-round infectivity experiments above, there is sufficient time for new viruses released by the cells to infect other cells. In these simulations, we assume that permissive or non-A3G expressing CEM-SS cells are used, with *t_T_*_,2_ = 30 hours (*k_p_* = 0.5545 day^−1^) [71], [72]. In the multicellular model, we focus on the release of new HIV. This begins approximately 0.9 days post infection [53], [57], [69], so *t*_prod_ = 22 hours. Also, on average a life-span of 2.3 days was estimated for infected cells [53], therefore *t*_dead_ = 55 hours. In exploring the multicellular model, we assume that the release rate of viruses is constant during the productive phase. Later, when we integrate the single-cell model, the actual time-varying distribution of virus release is employed. Several estimates using different techniques are available, ranging from a hundred to a few thousand viruses per cell [73]–[79]. HIV clearance rate *in vivo* has been estimated in the range of a few hours to a couple of days [53], [80]–[82], however, measurements of HIV clearance rate *in vitro* are not available. Using experimental data in [11], we estimate *k*_inf_, *B*, and *t_v_*_,1/2_. In [11], CEM-SS cells (*T*_0_ = 500,000) were stably co-transfected with either an A3G- or *neo*-encoding vector. Then, both cell lines were inoculated by either ΔVif or WT viruses with an initial dose of 1 or 10 ng p24. Accumulation of p24 in the culture supernatants was monitored over time. As expected, efficient replication of both WT and ΔVif viruses was observed in the *neo*-expressing cells. The A3G-expressing cells also supported WT virus growth, however, very low replication of ΔVif viruses was observed.

**Figure 6:**
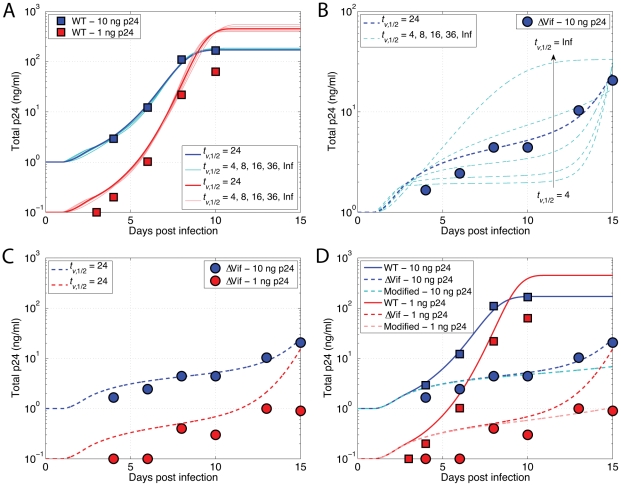
HIV growth curves for WT and ΔVif viruses. (A) Inoculation of cultures of 500,000 cells with WT HIV. Blue and red squares represent 1 and 10 ng p24 HIV input, respectively. For a given *t_v_*_,1/2_, values of burst size and virus infectivity rate were estimated such that the resulting simulated HIV growth curve fitted the blue data point with the minimum fitness error (shown as blue lines for *t_v_*_,1/2_ = 4, 8, 16, 24, 26, Inf hours). Then, the estimated numbers were used to predict experimental data points corresponding to 1 ng p24 (shown as red lines). (B) Inoculation by 10 ng p24 ΔVif HIV. For each triplet of (*t_v_*_,1/2_, *B*, *k*_inf_) from (A), the values of *p* and *c* between 0 and 1 were chosen such that the generated curve provided the smallest error. None of the values of *t_v_*_,1/2_ produced good fits to the blue circles except *t_v_*_,1/2_ = 24 hours where *p* and *c* were estimated to be 0.008 and 0.008, respectively. (C) The estimated parameters for *t_v_*_,1/2_ = 24 hours from (A) and (B) were used to examine how well they could generate a curve to fit experimental data points corresponding to 1 ng p24 ΔVif input (red circles). The red dashed line provided an acceptable fit to the data points except for the last circle where the line diverged. (D) All the experimental data points as well as their HIV growth curves are shown in red and blue colors corresponding to 1 and 10 ng p24 HIV input. Also, we included crowding effects in our simulation by using a logistic function. The two new curves drawn in light red (1 ng p24 ΔVif) and light blue (10 ng p24 ΔVif) show the HIV growth curves for this case. It can be seen that these curves provide a better fit to the experimental data than the curves in (C).

Since WT HIV replication in both *neo*- and A3G-expressing cells were almost the same, we conclude that the amount of A3G was insufficient to have a large effect on WT HIV replication, suggesting that Vif had completely inhibited A3G and most of newly produced viruses did not contain A3G. Therefore, even though we have A3G-expressing cells, we assume *p* = 1. Hereafter, we dismiss *neo*-expressing cells and only focus on A3G-expressing cells. Data points taken from the published experiments describing the increase in WT HIV numbers *in vitro* are shown in Fig. 6A with red and blue squares representing 1 and 10 ng p24 input HIV, respectively. As seen in Fig. 6A, data points corresponding to 1 ng p24 start at 0.1 ng p24/ml on day 3 after inoculation of cell culture. This means that the volume of cell culture was equal to 10 ml. In our simulations, the blue data points (10 ng p24) are used as training data for parameter estimation. Then, we change the initial dose to 1 ng p24 while keeping the estimated parameters fixed and run the model to examine how well it can reproduce the red data points.

**Figure 7:**
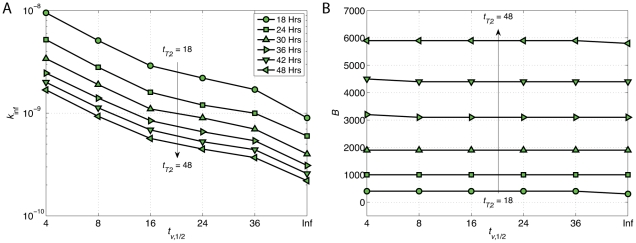
Effects of HIV half-life and cell doubling time on virus infectivity rate and burst size. (A) *k*_inf_ decreases as *t_T_*_,2_ changes from 18 to 48 hours. The same trend is also observed as *t_v_*_,1/2_ increases from 4 hours to infinity. (B) Estimated burst size remains almost the same for different values of *t_v_*_,1/2_, however, it increases as *t_T_*_,2_ goes up.

Given a specific value for viral half-life *t_v_*_,1/2_, we used the default nonlinear curve-fitting function in Matlab to find the optimum pair of burst size and infectivity (*B*, *k*_inf_) such that the simulated HIV growth curve fits the experimental data (WT - 10 ng p24) with the minimum fitness error. This error is the discrepancy between experimental and computational results defined by square root of sum of squares of differences between experimental data and computed results at each observation time point. We studied the effects of HIV half-life and cell doubling time on these estimates of optimal *B* and *k*_inf_ in Fig. 7A and Fig. 7B. As *t_v_*_,1/2_ increases, the estimated value of *k*_inf_ decreases while estimated burst size stays roughly the same. However, as *t_T_*_,2_ changes from 18 to 48 hours, the estimates of *B* increase approximately from 400 to 5900 while the values of *k*_inf_ decrease less than an order of magnitude. This is reasonable in a sense that in order to get the same amounts of HIV output as *t_T_*_,2_ increases, viruses must infect with lower rates (*k*_inf_ ↓), however, they must produce more progeny in the cells (*B* ↑). Since we know *t_T_*_,2_ = 30 hours, only the optimum values of *B* and *k*_inf_ for each value of *t_v_*_,1/2_ are shown in Table 2.

**Table 2:** Estimations of burst size and virus infectivity rate for *t_T_*_,2_ = 30 hours.

*t_v_*_,1/2_ (hr)	*B*	*k*_inf_ (day^−1^)
4	1900	34×10^−10^
8	1900	19×10^−10^
16	1900	11×10^−10^
24	1900	9×10^−10^
36	1900	7×10^−10^
Inf	1900	4×10^−10^

For each set of parameters in Table 2, simulated HIV growth curves are shown in Fig. 6A with dark blue representing *t_v_*_,1/2_ = 24 hours and light blue representing other values for *t_v_*_,1/2_ in the range of 4 hours to infinity. The curves overlap and are a good fit for the blue squares. HIV growth curves with the same parameters and 1 ng p24 HIV input are also depicted in Fig. 6A with dark (*t_v_*_,1/2_ = 24) and light (*t_v_*_,1/2_ = 4 … Inf) red colors. Again, it is observed that all the curves are similar and provide a good fit for the red data points, with the exception of the last square, which we will discuss later. Therefore, none of the tested values for viral half-life, *t_v_*_,1/2_, can be dismissed at this point, since all of them have generated good fits for the experimental data.

Now, we focus on the experimental data obtained from 10 ng p24 ΔVif viruses (blue circles in Fig. 6B). HIV growth curves corresponding to different values of *t_v_*_,1/2_ increasing from the lowest curve to the highest one are also shown in Fig. 6B. These curves were generated as follows. For each triplet of values for (*t_v_*_,1/2_, *B*, *k*_inf_) from Table 2, the optimal values of *p* and *c* between 0 and 1 were chosen using Matlab's built-in nonlinear curve-fitting function such that the generated curve provided the lowest error (deviation from experimental data). Although all the values of *t_v_*_,1/2_ could provide a good fit to the experimental data points in Fig. 6A, they all failed to produce good fits to the blue circles in Fig. 6B except *t_v_*_,1/2_ = 24 which provided a good match with *p* = 0.008 and *c* = 0.008. Therefore, we conclude that HIV half-life *in vitro* is approximately 24 hours and we use this number and corresponding numbers from Table 2 for burst size and infectivity rate in the rest of this study. Note that these are the optimal values of *t_v_*_,1/2_, *B*, and *k*_inf_ that were used in multicellular model in the last section where we estimated *P_Vif_* and *P_A3G_*.

In Fig. 6C, red circles correspond to 1 ng p24 ΔVif input and a simulation curve using the estimated parameters is superimposed. This curve is a reasonable fit to the data points except for the last red circle. Compared to the 10 ng p24 data, the experimental data points for 1 ng p24 ΔVif input are noisy, possibly because of the small amounts of p24 in culture supernatant which are initially close to the detectable level of the p24 ELISA assay.

**Figure 8:**
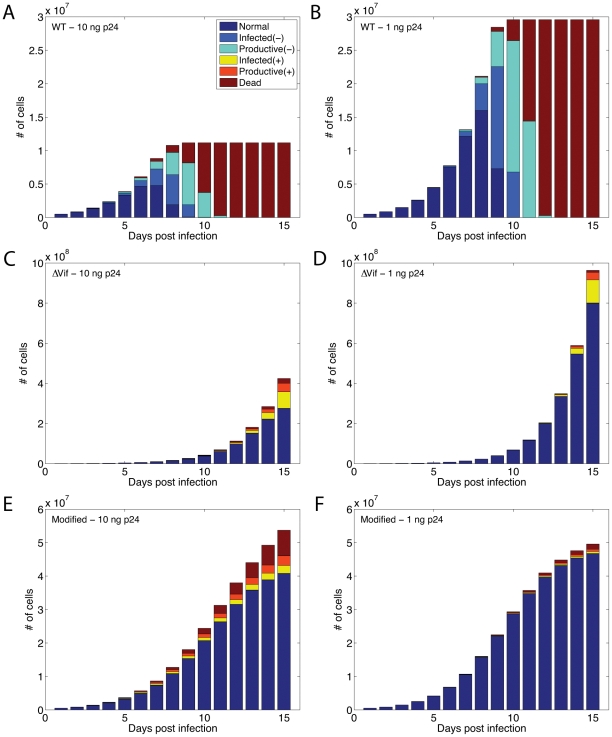
Distribution of cell states during the period of post infection. We simulated cultures of 500,000 healthy normal cells inoculated by either (A) 10 ng p24 or (B) 1 ng p24 WT HIV input. The infected cells start producing new virions after 22 hours and eventually die around 55 hours after infection. For WT HIV input, most of the cells are dead by the 12th day. In contrast, if we inoculate the cultures with either (C) 10 ng p24 or (D) 1 ng p24 ΔVif viruses, the healthy cells will still be the majority ones and the number of dead cells is negligible on the 15th day. In a different scenario, we included effects of cell culture crowding in our multicellular model by using a logistic function. Such cultures inoculated with either (E) 10 ng p24 or (F) 1 ng p24 ΔVif viruses provide better fits to biological experiments.

At this point, we must ask: why do the simulated HIV growth curves saturate around 9–10 days after infection in the case of WT viruses? In order to explain this, we study the distribution of cell states during the period of post infection. As seen in Fig. 8A for the case of 10 ng p24 WT HIV input, normal cells dominate from the beginning of infection until the 8th day. But as infection progresses, infected cells begin to take over. These cells begin production of new viruses at around 22 hours after infection so the dominant cells on the 9th day are productive cells. Finally, viruses kill the productive cells at around 55 hours post infection and thus after the 11th day, almost all the cells in the culture are dead. Therefore, no more viruses can be produced and the HIV growth curve plateaus. The same scenario is observed in Fig. 8B (WT – 1 ng p24), however, infection progresses more slowly due to presence of fewer viruses initially. As seen in Fig. 8B, the number of normal cells is much higher than the previous case in the period of days 1–9. This explains why the total number of viruses will eventually be higher than the case of 10 ng p24 WT in Fig. 6A. The reason is that many more normal cells are available for HIV infection in the case of 1 ng p24 WT, which results in production of more viruses in the model. This is a testable prediction of the model, which would require the experiment to be continued after 10 days.

Looking at Fig. 8D for ΔVif viruses, we see more than 80 percent of the cells are healthy on the 15th day and the percentage of dead cells is negligible, in clear contrast to what was seen for WT viruses in Figs. 8A–B. This is because of the less efficient propagation of infection of ΔVif viruses allowing normal proliferation of healthy cells. This in turn provides yet more cells for HIV to infect compared to the WT case. In fact, it may explain the rise of the ΔVif growth curve in Fig. 6C and its divergence from the experimental data points. In our model, we have assumed constant cell proliferation rates, however, cells might slow or even stop proliferation when the suspension becomes crowded. In order to test whether this was a possible explanation for the observed data, we included the crowding effects in our model by using the logistic function. Therefore, (7) is replaced by
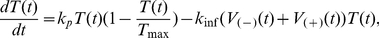
(14)where *T*_max_ is the maximum possible number of normal cells in culture and we set it to 50,000,000 (5,000,000 cells/ml). Modified ΔVif growth curves are shown in Fig. 6D with light blue and red colors for 10 and 1 ng p24 ΔVif input, respectively. These curves show better fits to the experimental data, suggesting that crowding effects and slow proliferation could explain the experimental results. The distributions of cells states corresponding to these two cases are shown in Figs. 8E–F.

### Effects of A3G-Based Therapeutic Strategies, Drug Penetrance and Administration Time on HIV Replication

Using the model parameterized as above, we can compare the predicted efficacy of several therapeutic approaches targeting Vif-A3G interactions. Here, we add four specific molecules to the model and simulate the effect of their intracellular expression. All four are large proteins, as opposed to small molecules, and expression would in most cases require gene therapy. However, small molecules that had similar properties or effects on the functional A3G-Vif network could be delivered orally or intravenously [83], [84]. The molecules are: **Ab-Vif**, a high-affinity antibody to Vif [85]; **A3G**, APOBEC3G itself, which could be upregulated by cytokines such as IL-2 [86] or NFAT and IRF proteins [87]; **A3G^ΔUb^**, a mutated A3G with lower Vif-induced degradation rate (e.g., C97A-A3G [88]); and **A3G^ΔVif^**, a mutated A3G that does not bind Vif (e.g. A3G/F126-129 [89] and D128K-A3G [90]).

**Figure 9:**
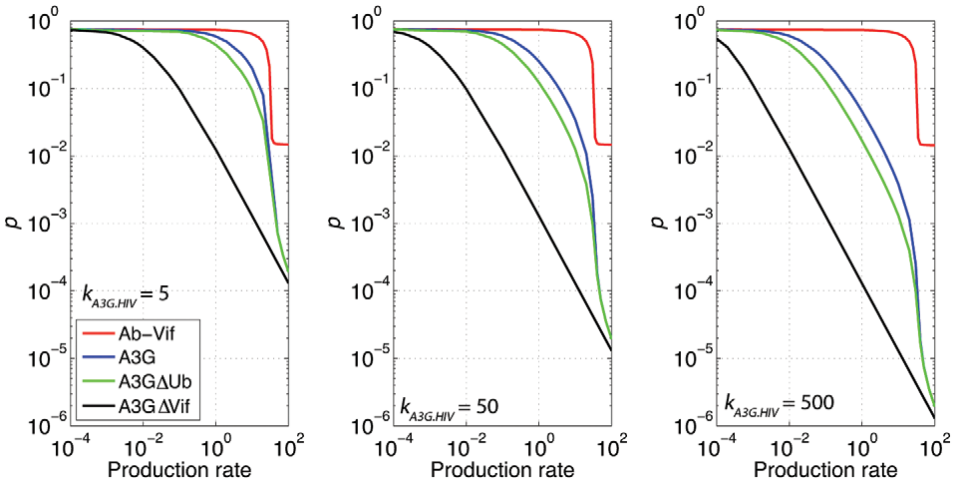
Efficacy comparison of several drugs for different production rates. Efficacy of several proposed therapeutic proteins in reducing the parameter *p* estimated using the single-cell model for (A) *k_A3G.HIV_* = 5, (B) 50 and (C) 500 µM^−1^/hr. For all cases, A3G^ΔVif^ shows a better performance than other drugs.

In the single-cell model, Ab-Vif is modeled as a new protein with an affinity for Vif 100 times that of A3G. The degradation rate of the complex formed by antibody bound to Vif is assumed to be *k*_d,*Vif*_. Upregulation of A3G is modeled by increasing *P_A3G_*. A3G^ΔUb^ is a mutated A3G that binds to Vif, but its complex with Vif is not degraded faster than unbound A3G. Therefore, the degradation rate of A3G^ΔUb^-Vif complex is assumed to be *k*_d,*A3G*_. Finally, A3G^ΔVif^ has the binding site for Vif mutated, and so does not bind to Vif. Note that we also assumed that each of these therapeutic proteins has the same degradation rate as of A3G. In our simulations, each of the drugs is expressed *in addition* to the cellular A3G produced (at a rate of *P_A3G_*). The efficacy of each drug in terms of reduction in HIV replicative potential versus various production rates is shown in Fig. 9 for *k_A3G.HIV_* = 5, 50 and 500 µM^−1^/hr. Among the therapeutic approaches, Ab-Vif shows a very poor performance even at very high production rates. It should be noted that Ab-Vif on its own can only block Vif from binding to A3G; in other words, it can make A3G more available but cannot add to its function. At least some A3G must be present in the cells to get incorporated into HIV particles. This explains the characteristic plateau as Ab-Vif expression increases (Fig. 9); beyond this point all A3G is available to be encapsulated. A3G and A3G^ΔUb^ have efficacy profiles that are similar to each other, both better than that of Ab-Vif. That A3G^ΔUb^ is predicted to work only slightly better than A3G suggests that Vif-induced degradation of A3G through proteasomal pathway is not central to Vif effectiveness. A3G^ΔVif^ is predicted to be the best therapeutic approach among these drugs, two orders of magnitude better than A3G and A3G^ΔUb^. This further suggests that the binding of A3G to Vif is an important interaction that should be inhibited to block HIV replication.

**Figure 10:**
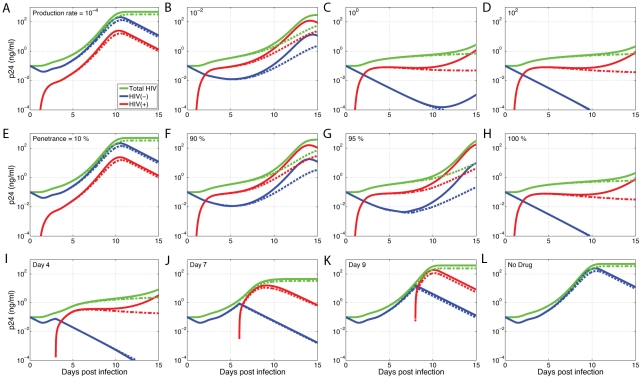
Effects of different production rates, penetrances and administration times of A3G^ΔVif^ on HIV growth curve. In all the simulations, 500,000 cells were inoculated by 1 ng 24 WT HIV input. (A–D) A3G^ΔVif^ with different production rates were administered right after inoculation. The red and blue lines represent A3G(−) and A3G(+) viruses in the culture, respectively. The green lines characterize all the viruses including A3G(−), A3G(+), and dead ones. For *P_A3GΔVif_* = 10^2^, the amount of A3G(−) viruses decay to 10^−4^ ng p24/ml by the 10th day, however, the number of A3G(+) viruses rises on the 12th day. Dashed lines represent cultures with constrained proliferation (crowding effects modeled by using a logistic function). In this case, it is seen that A3G(+) viruses reach a stable level below 10^−1^ ng p24/ml and decrease very slowly up to the 15th day for *P_A3GΔVif_* = 10^2^. This suggests that A3G^ΔVif^ has been able to stop HIV replication. (E–H) Effects of drug penetrance on HIV growth curves. We simulated cases where the drug was only available to a fraction of cells (*P_A3GΔVif_* = 10^2^). Comparing cases corresponding to 95% and 100%, we can see that there is a gap larger than two orders of magnitude between the total levels of p24 on the 15th day. This implies that drugs should be available to all the cells to get the desired efficacy. The same qualitative effect is observed in the cultures with constrained proliferation for different drug penetrances. (I–L) Effects of administration time on HIV growth curves (*P_A3GΔVif_* = 10^2^ and penetrance = 100%). It is seen that administering drug on the 9th day is not effective and the results are similar to the case of no drug. However, if the drug is administered before the 7th day, cell could still survive. The same trend in effects of drug administration time is also observed in cultures with constrained proliferation.

We next study A3G^ΔVif^ in the multicellular model, assuming 1 ng p24 WT input and *k_A3G.HIV_* = 50 µM^−1^/hr, although qualitatively similar results will be obtained for other values of HIV inputs and *k_A3G.HIV_* (data not shown). The intracellular and multicellular models were coupled using (12a) and (12b) to compute the total number of A3G(−) and A3G(+) viruses in culture supernatant. Figs. 10A–D show HIV growth curves corresponding to various production rates of A3G^ΔVif^. The blue and red lines represent A3G(−) and A3G(+) viruses, respectively, whereas, the greens lines represent total viruses in culture supernatant including A3G(−), A3G(+), and dead ones. As seen in Fig. 10D, for *P_A3GΔVif_* = 10^2^, HIV replication slows more than two orders of magnitude and A3G(−) viruses reach a level of 10^−4^ by the 10th day. However, A3G(+) viruses start boosting by the 12th day. As mentioned earlier, this is caused due to an unconstrained proliferation of normal cells that provides a huge number of susceptible cells for infection. Similar to before, we constrained proliferation by including crowding effects using a logistic function (dashed lines in Fig. 10). In this case, A3G(+) viruses reach a stable level below 10^−1^ ng p24/ml and decrease very slowly up to the 15th day. Therefore, A3G^ΔVif^ has actually been able to stop HIV replication. A comparison of model predictions using equations (11a/b) or (12a/b) as the coupling method is shown in Supplemental Fig. S1.

At this point, we are interested to study the effects of drug penetrance on HIV replication when only a specific fraction of cells have been transfected (*P_A3GΔVif_* = 10^2^). Figs. 10E–H show HIV growth curves with penetrance = 10, 90, 95 and 100% of cells. It is observed that even if the drug is available to 95% of the cells, viruses can still actively replicate until they kill all the cells in the solution. This suggests that the drug must be available to almost 100% of the cells in order to be effective. HIV growth curves with similar penetrances for the constrained proliferation case are also depicted in Figs. 10E–H. Even in this case, there is a large gap between curves corresponding to 95 and 100% drug availability.

In the next set of simulations, we studied the effects of drug administration time on the virus replication (*P_A3GΔVif_* = 10^2^, penetrance = 100%). As seen in Fig. 10K, if the drug is administered on the 9th day, the HIV growth curve is almost similar to the case that no drug was available to the cells at all as shown in Fig. 10L. Note that these results were obtained for *in vitro* cases, where a constant source of cell production is not available as opposed to *in vivo* cases where old cells proliferate and new cells are born. Also, it should be mentioned that more than 50% of the cells are either infected or productive on the 9th day (Fig. 8B) and a lot of viruses are available in culture supernatant. Therefore, administration of drug to remaining cells cannot help the culture survive. However, cells in the culture can still survive if we administer the drug before the 7th day. This suggests that the drug must be available to the cells shortly after inoculation in order for the drugs to be effective. *In vivo*, the situation would be different; the constant birth of new cells may give this therapy greater hope of success.

### Sensitivity Analysis: Determining Critical Model Parameters that Influence HIV Replication

**Figure 11:**
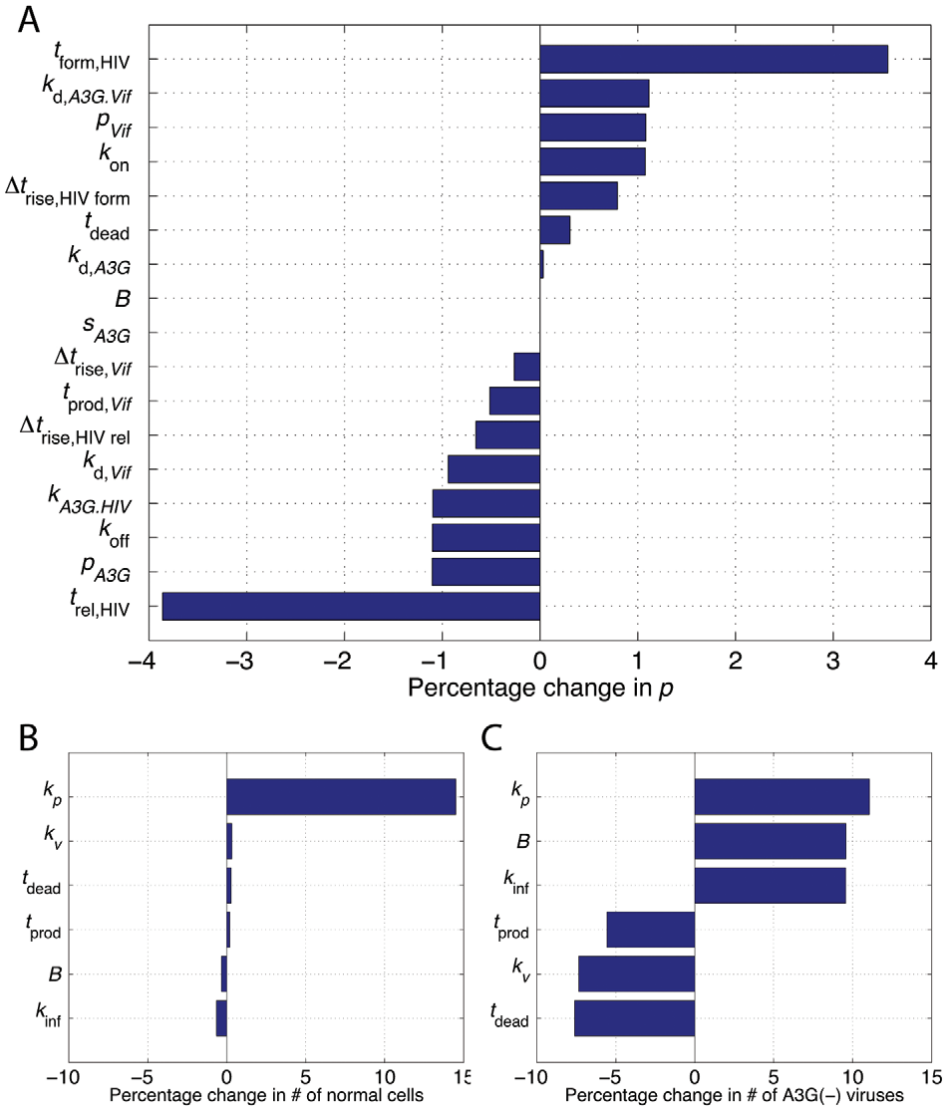
Sensitivity analysis in single-cell and multicellular models. (A) The values of all 17 parameters in the intracellular model have changed by +5% and the percentage change in *p* for each of them is shown. Two parameters representing the time origins of virus release and particle formation had the highest impact on *p*. In contrast, parameters such as burst size, the degradation rate of A3G, and the stoichiometry of A3G proteins incorporated in HIV particles had zero or negligible effects on *p*. For the extracellular model, the effects of parameter variations were studied on two outputs; (B) number of normal cells and (C) number of A3G(−) viruses on the 6th day. In both cases, variations of cells proliferation rate had the highest impact on the extracellular model outputs.

In this section, we analyze the effects of parameter variations in both single-cell and multicellular models. For the intracellular model, we investigate the deviations of HIV replicative potential resulted from +5% change in each of the 17 model parameters (Fig. 11A). As seen, *t*_form,HIV_, the particle formation starting time, had the highest positive impact on *p*, whereas *t*_rel,HIV_, the virus release starting time, had the highest negative impact. This suggests that if the assembly and budding process of HIV particles from the cells could somehow be slowed, it would have a significant effect on virus replication. On the other hand, some of the parameters such as *s_A3G_*, burst size, and *k*_d,*A3G*_ had very negligible effects on the intracellular model output.

For the extracellular model, two outputs are considered for sensitivity analysis; 1) number of healthy cells and 2) number of A3G(−) viruses on the 6th day. In our simulations, 1 ng p24 WT HIV input was used for infection without administering any drug. In terms of the 1st output, almost none of the parameters had a major impact except the proliferation rate, *k_p_*, which produced high variations in the number of normal cells (Fig. 11B). In contrast, all the multicellular parameters had significant effects on the levels of A3G(−) viruses (Fig. 11C). Considering the combined effects on both outputs, *k_p_* was detected as the most sensitive parameter in the multicellular model.

## Discussion

We have developed a mathematical model of the HIV lifecycle inside and outside of cells, using differential equations. Our model is the first one developed to specifically couple molecular-level events within individual cells to the viral dynamics and multiple cycles of infection within a population of cells. In this paper, we used two different methods to couple the two models. Estimation of the system parameters was done using the model with equations (11a) and (11b) in which the release rate of viruses was assumed to be constant over period of [*t*_prod_, *t*_dead_] and also *p* remained constant during this period. For the rest of our simulations to study effects of A3G-based therapies, the model with equations (12a) and (12b) was used in which the time-dependent distribution of virus release from a single cell was employed to compute the total number of A3G(−) and A3G(+) viruses. The multi-scale system allowed us to achieve a quantitative understanding of the Vif-A3G pathway in HIV pathogenesis. Experimental data were used to establish system parameters such as stoichiometry of molecules, degradation rates of proteins, production profiles of viral proteins, viral burst size, cell proliferation rate, life-span of infected cells, viral generation time and virus clearance rate. We validated our system by reproducing the results of *in vitro* T cell culture experiments. We found that both downstream effects of A3G (hypermutation and reduction of viral burst size) were important to replicate the experimental results *in silico*. Based on the model simulations, *in vitro* virus clearance was estimated to be 24 hours. The model also predicted that the average number of HIV viruses produced by an infected cell is 1900. We simulated two types of T cell cultures with unconstrained or constrained proliferation rate (including crowding effects by using a logistic function). It was observed that simulated HIV growth curves provided better fits to the experimental data in the latter case suggesting that proliferation may slow down in cell culture after it gets crowded.

Several therapeutic molecules targeting the Vif-A3G pathway were tested in our system. These included a high-affinity antibody to Vif [85], APOBEC3G itself, a mutated A3G with lower Vif-induced degradation rate (A3G^ΔUb^) [88], and a mutated A3G that does not bind Vif (A3G^ΔVif^) [89], [90]. It was found that A3G^ΔVif^ was the most effective drug that could stop HIV replication. This also implied that inhibition of A3G binding to Vif is a crucial step in blocking HIV replication. We further studied A3G^ΔVif^ with respect to effects of penetrance and administration time on HIV replication. The model predicted that the drug must be available to almost 100% of the cells in order to get the desired efficacy. Also it must be available to the cells shortly after inoculation in order for the cells to survive.

Sensitivity analysis of the single-cell and multicellular models helped us characterize parameters with significant impacts on the system. We did a local sensitivity analysis by changing each parameter by 5% and study their effects on the output parameters. In the single-cell model, we chose HIV replicative potential, the ratio of released A3G(−) viruses to the total number of released viruses, as the output parameter. This is a critical parameter in our system linking the two models together. We found that *t*_rel,HIV_ and *t*_form,HIV_ are the most sensitive parameters. This implies that slowing the assembly and budding process of HIV particles from the cells reduces the number of output A3G(−) viruses. In the multicellular model, two outputs were chosen for sensitivity analysis: 1) number of healthy cells and 2) number of A3G(−) viruses on the 6th day. We found that the proliferation rate of cells had the highest combined impact on both output parameters.

In this study, we primarily focused on molecular and cellular processes of HIV infection *in vitro*, however, this provides the necessary requirements to expand the model and move towards *in vivo* computation modeling of HIV. In the extended model, virus clearance *in vivo* and the mechanisms of cell birth, proliferation, and death would be different and new topics such as latency would come into play. Also, CD4+ T cells in the immune system can function as memory cells. Therefore, they can latently carry integrated HIV for the duration of their lifetime. These cells can survive for years and possibly decades and upon withdrawal of antiretroviral therapy, they become active and HIV viral loads rebound quickly. So, this concept of latency stage should also be accounted for in the model by having a small population of dormant infected cells that live for a long period of time and infrequently become activated to produce HIV. In addition, the immune system is also hugely diverse and has many more cells than *in vitro* cell culture experiments. Also, some tissues such as GI tract are more susceptible to HIV infection than others. Therefore, compartmentalization is essential and specific models need to be developed for each tissue and they must be closely linked to represent the whole body. The *in vivo* model will be more complicated but can answer some more fundamental questions about HIV pathogenesis than we can not cover with *in vitro* modeling.

## Supporting Information

Figure S1 **Comparison of model predictions using the two coupling methods.** In all the simulations, 500,000 cells were inoculated by 1 ng 24 WT HIV input. A3G_ΔVif_ with different production rates were administered right after inoculation. The red and blue lines represent A3G(−) and A3G(+) viruses in the culture, respectively. The green lines characterize all the viruses including A3G(−), A3G(+), and dead ones. Dashed lines represent cultures with constrained proliferation (crowding effects modeled by using a logistic function). The intracellular and multicellular model were coupled using either (A–D) equations (12a/b) or or (E–H) equations (11a/b). Although the coupling method using equations (11a/b) assumes that release rate of viruses from a productive cell (and the ratio of A3G(−) to total viruses) is constant over the period [**t**_prod_, **t**_dead_], it provides a very good approximation of the model predictions obtained by the second coupling method using equations (12a/b). Note that there is no assumption on the rate of virus release in the second method and the actual time-dependent profile of virus release from a single cell is used to compute the total number of A3G(−) and A3G(+) viruses in culture supernatant.
(PDF)
