# Supplementary material for: Multi-Scale Modeling of HIV Infection in vitro and APOBEC3G-Based Anti-Retroviral Therapy
Source: PLoS Comput Biol. 2012 Feb 9;8(2):e1002371. doi: 10.1371/journal.pcbi.1002371 (PMC3276540; doi:10.1371/journal.pcbi.1002371)
Supplement: Figure S1 — Comparison of model predictions using the two coupling methods. In all the simulations, 500,000 cells were inoculated by 1 ng 24 WT HIV input. A3GΔVif with different production rates were administered right after inoculation. The red and blue lines represent A3G(−) and A3G(+) viruses in the culture, respectively. The green lines characterize all the viruses including A3G(−), A3G(+), and dead ones. Dashed lines represent cultures with constrained proliferation (crowding effects modeled by using a logistic function). The intracellular and multicellular model were coupled using either (A–D) equations (12a/b) or or (E–H) equations (11a/b). Although the coupling method using equations (11a/b) assumes that release rate of viruses from a productive cell (and the ratio of A3G(−) to total viruses) is constant over the period [t prod, t dead], it provides a very good approximation of the model predictions obtained by the second coupling method using equations (12a/b). Note that there is no assumption on the rate of virus release in the second method and the actual time-dependent profile of virus release from a single cell is used to compute the total number of A3G(−) and A3G(+) viruses in culture supernatant. (PDF) [file pcbi.1002371.s001.pdf]

Multi-Scale Modeling of HIV Infection *in vitro* and APOBEC3G-Based Anti-Retroviral Therapy

Iraj Hosseini and Feilim Mac Gabhann

Supplemental Figure

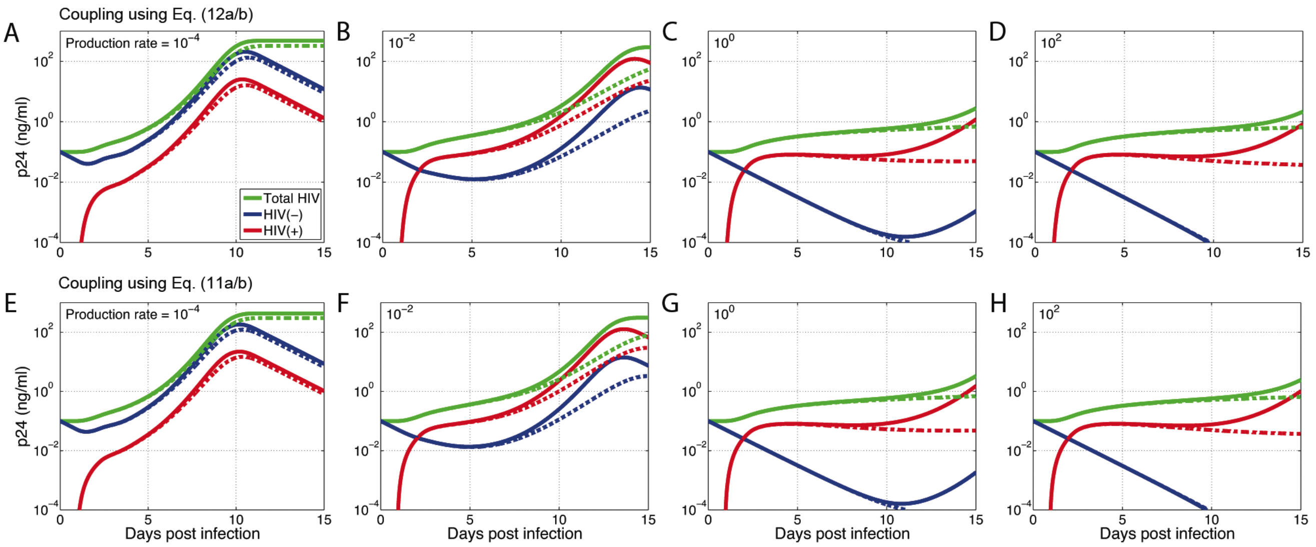

Figure S1. Comparison of model predictions using the two coupling methods. In all the simulations, 500,000 cells were inoculated by 1 ng 24 WT HIV input. A3G$^{\Delta Vif}$ with different production rates were administered right after inoculation. The red and blue lines represent A3G(-) and A3G(+) viruses in the culture, respectively. The green lines characterize all the viruses including A3G(-), A3G(+), and dead ones. Dashed lines represent cultures with constrained proliferation (crowding effects modeled by using a logistic function). The intracellular and multicellular model were coupled using either (A-D) equations (12a/b) or or (E-H) equations (11a/b). Although the coupling method using equations (11a/b) assumes that release rate of viruses from a productive cell (and the ratio of A3G(-) to total viruses) is constant over the period [t_{prod}, t_{dead}], it provides a very good approximation of the model predictions obtained by the second coupling method using equations (12a/b). Note that there is no assumption on the rate of virus release in the second method and the actual time-dependent profile of virus release from a single cell is used to compute the total number of A3G(-) and A3G(+) viruses in culture supernatant.
